# SED, a normalization free method for DNA microarray data analysis

**DOI:** 10.1186/1471-2105-5-121

**Published:** 2004-09-02

**Authors:** Huajun Wang, Hui Huang

**Affiliations:** 1 Oscient Pharmaceuticals Corporation, 100 Beaver St, Waltham, Massachusetts 02453, USA

## Abstract

**Background:**

Analysis of DNA microarray data usually begins with a normalization step where intensities of different arrays are adjusted to the same scale so that the intensity levels from different arrays can be compared with one other. Both simple total array intensity-based as well as more complex "local intensity level" dependent normalization methods have been developed, some of which are widely used. Much less developed methods for microarray data analysis include those that bypass the normalization step and therefore yield results that are not confounded by potential normalization errors.

**Results:**

Instead of focusing on the raw intensity levels, we developed a new method for microarray data analysis that maps each gene's expression intensity level to a high dimensional space of SEDs (Signs of Expression Difference), the signs of the expression intensity difference between a given gene and every other gene on the array. Since SED are unchanged under any monotonic transformation of intensity levels, the SED based method is normalization free. When tested on a multi-class tumor classification problem, simple Naive Bayes and Nearest Neighbor methods using the SED approach gave results comparable with normalized intensity-based algorithms. Furthermore, a high percentage of classifiers based on a single gene's SED gave good classification results, suggesting that SED does capture essential information from the intensity levels.

**Conclusion:**

The results of testing this new method on multi-class tumor classification problems suggests that the SED-based, normalization-free method of microarray data analysis is feasible and promising.

## Background

DNA microarray technology is now playing an increasingly important role in biomedical research. Microarray technology gives one the opportunity to measure gene expression levels of thousands to tens of thousands of genes simultaneously, in order to study the differential gene expression pattern between different developmental stages, diseases states and samples treated with drugs or other compounds. Before comparing data from different arrays to address these biological questions, however, a "much more mundane but indispensable " normalization step [1] is currently used in most microarray analyses.

Because of the slight difference in RNA quantities, imaging settings and other variables, even in very controlled experiments the intensity levels from different arrays are of different scales and need to be normalized before they can be compared with each other. Various normalization methods have been developed and some are widely used. The simplest method is total intensity based normalization [2]; this approach scales intensity levels of every gene by a constant factor so that total intensities of all the arrays are the same. "Spiked-in" based normalization methods scale intensity based on spiked-in standards [3]. Nonlinear normalization methods use local regression to scale intensities to compensate for the intensity-dependent differences between arrays [4-6].

For most current applications, these normalization methods seem to be adequate. However, the residual left by a less than perfect normalization procedure is another source of non-biological variation that is usually non-desirable, especially when the differences in expression levels are expected to be small [7]. In addition, if the goal is meta-analysis of multiple sets of microarray data [8,9] systematic differences between experiments may result in a normalization artifact. We were therefore interested in developing an approach to analyse microarray data without first performing a normalization step. Our approach was partly inspired by non-parametric statistical methods [10]. For example, nonparametric methods that use ranks [11,12] to compare microarray results, in addition to being distribution free, have the additional advantage of being normalization free.

DNA microarray technology has been used widely in biomedical studies. One interesting application is in the area of molecular classification; one popular use is in the comparison of tumor samples. Since clinical and histopathological classification is sometimes difficult and labor-intensive, the use of genome wide expression patterns to classify tumor samples has recently become a very active research area [13-16]. Although some tumors appear to be amenable to classification using microarray data [17,18], general multiple tumor classification using microarray data has proved to be an interesting and challenging task for several reasons: the general difficulties inherent in multi-class classification problems, the small number of samples available, and the inherent biological variation between specimens, *etc*. We decided to use multi-class tumor classification as a test case to illustrate the power of our approach. We compared our results for a multi-class tumor classification problem with more conventional approaches published by Ramaswamy *et al*. [19] and Yeang CH *et al. *[20]. These authors compared the accuracies of using k-Nearest Neighbors (kNN, 60–70%), Weighted Voting (WV, 60–70%) and Support Vector Machine (SVM, 80%) algorithms in a multi-class tumor classification problem and concluded that SVM is a more powerful machine learning algorithm for this application.

## Results

### Normalization Free approach to microarray data analysis

Generally, measurements on single microarrays give a real-valued intensity level x_i _(1<= i <= N) for each gene i on the array, where N is the total number of genes on the array. Without first doing some type of normalization, the intensity level of gene i from array A, x_i_^A^, cannot be directly compared with the intensity level of gene i from array B, x_i_^B^. In this study, we sought an alternative quantity or quantities that can be directly compared between different arrays without compromising important biological information. One obvious candidate is r_i_, the rank of intensity level of gene i on the array. However, we felt that rank is not an adequate measure because information about relative expression level is not represented explicitly. Instead, we decided to use the following measures.

Let

s_ij _= 1 if(x_i _- x_j _> 0)

0 otherwise     (1)

, where 1<= i, j <= N. Basically, s_ij _is the sign of the intensity difference of gene i and j on a single microarray and therefore will remain unchanged under any monotonic transformation of x. Therefore, instead of computing with the absolute expression level of a gene, its relative level to all the other genes on the microarray is used. For each gene i, instead of one real valued x_i_, the approach uses s_i _= (s_i1_, ..., s_ij_, ..., s_iN_), a binary vector of size N. For ease of reference, we will simply refer to this value as the SED (Signs of Expression Difference) of gene i; and the entire matrix (s_ij_) the SED of the array. Given (x_i_), s_ij _is simply and uniquely defined but (s_ij_) does not uniquely determine x_i _so some information is lost by only using (s_ij_) instead of (x_i_).

Since r_i _= Σ_j = 1,...,N _s_ij _is the rank of gene i in terms of intensity levels, rank information is preserved in (s_ij_). What is lost in the transformation from (x_i_) -> (s_ij_) is just the intensity differences between the closest ranked genes, which in most cases are small, considering that microarray data are generally considered "very noisy". It was our major goal to demonstrate that (s_ij_) has indeed captured important components of the information from (x_i_).

Instead of directly using the intensity levels x, and its derivatives such as the mean μ, the standard deviation σ and the signal to noise ratio (S2N) between two sample groups A and B, [(μ^A ^_+ _μ^B^)/(σ^A^+ σ^B^)], we will use (s_ij_) to compare gene expression differences between arrays. Since we expect measurement variations within an array will be less than those between arrays and we take the signs of relative expression differences to get SED, we expect the SED will be less "noisy". However, the value of any single s_ij _may still vary between technical and biological replicates. One would expect more s_ij _would change values randomly if the technical replicates was done on arrays that were fabricated in a different run than arrays from same run, for example. Biological variations are expected to be even more frequent. However, we hypothesize that we can perform statistical analysis on the SED, which contains tens of thousands of s_ij _for a single gene, and minimize the impact of such noises.

We will also consider (sp_ij_), a natural generalization of the SED concept. Here, sp_ij _is the probability of x_i _> x_j_. In other words, imagining one can get a large number, n, of either technical or biological replicates of the sample of interest, then sp_ij _= m/n as n -> 8, where m = Σ_K = 1,...,n _s_ij_^K ^and s^K ^is for replicate K. We will call (sp_i_) = (sp_i1_, ..., sp_ij_, ..., sp_iN_) the SED probabilities of gene i. Note that in calculating both SED and SED probabilities, only intensity comparisons within arrays are involved and therefore forego the normalization step.

For example, if gene i is more highly expressed in sample A than B we would expect that more s_ij_^A ^than s_ij_^B ^would be 1 instead of 0 and the overall (very loosely defined) sp_ij_^A ^would be larger than sp_ij_^B^. Since rank can be calculated from SED, any rank based method can be expanded to use SED. A gene i's SED can be viewed in two different perspectives. On one hand, it provides information about gene i's expression level relative to every other gene on the array, and therefore can be used to examine gene i's expression patterns between samples. On the other hand, it also provides information about the expression levels of all the other genes on the array, using the gene i as a control, in essence. Therefore, SED can be used to study questions either at the gene level or at the array level. In this paper, we focus on solving a simpler problem at the array level where it is not necessary to decide whether the expression level of an individual gene is increased or decreased between array A and B and by how much. Rather, it is focused on whether the overall expression patterns are different at all between array A and B.

### Multi-class classification of tumor samples

To test whether (s_ij_) and (sp_ij_) extracts most of the information from (x_i_), we used these values in a test case of a multi class classification problem described by Ramaswamy *et al. *and Yeang *et al *[19,20]. Two algorithms were used to classify each of the 144 tumor samples into one of 14 tumor classes. One is the Naive Bayes (NB) classifier [21] using SED probabilities. The other is the Nearest Neighbor (NN) classifier using SED. In the NB method, to classify a sample T, we first calculate (sp_ij_^C^) for each class C (1 <= C <= 14) using training samples, i.e. the 144 samples with T taken away (for details see methods). Then sample T is classified according to:

score(T, C)_= _Σ_i = 1,...N _Σ_j = 1,...,N _log(p_ij_),     (2)

where p_ij _= sp_ij_^C ^if s_ij_^T ^= 1

1 - sp_ij_^C ^otherwise

T is simply classified to the class C that has the maximum score.

In the NN method, we compute instead, for each training sample t,

matches(T, t) = Σ_i = 1,...N _Σ_j = 1,...,N _δ(s_ij_^T^, s_ij_^t^),     (3)

where δ(x, y) = 1 if x = y.

0 otherwise

Then T is classified to class C of the sample t that has the maximum matches.

If one is to give a statistical interpretation of these scores, one can simply view (sp_ij_) as defining a multi-binomial probability model. In addition, one could consider each s_ij _as a draw from a binomial distribution with probability p_ij _= sp_ij_. Then, the score(T, C) is simply a logarithm of the probability p to get all the s_ij _exactly the same as s_ij_^T ^under the probability model C where p_ij _= sp_ij_^C^. (Since each class defines a different probability model, score(T,C) for difference class C, in theory, should not be directly compared. Instead, a P value should be calculated from the probability model for Pr(p<exp(Score(T, C))) and used to evaluate the closeness of sample T to each class C. For simplicity, we are not considering such issues here.)

When the NB algorithm was applied to the 144 samples, the accuracy obtained was about 63%; the NN algorithm performed slightly better and gave an accuracy of 70%.

### Feature selection

Depending on the algorithm, a better classification result can sometimes be obtained by using a subset of genes [22,23]. We were interested to know whether feature selection helps to increase accuracy in our approach. Within our framework, it is easier to treat (i,j) pairs as selection units. We therefore filtered out (i,j) pairs where the variance of sp_ij _across the 14 tumor classes was less than a pre-determined value and left the rest of the algorithm unchanged. We reasoned that the filtered-out part of the matrix has less discriminating power across the tumor spectrum and might add noise due to the small sample sizes used. Using the NB algorithm, the best result achieved was 70% with a cutoff σ^2 ^= 0.06, while the NN method with σ^2 ^= 0.05 gave 77%. Table 1 lists the accuracies achieved using different feature cutoffs.

### "Single gene's SED" based classifier

The above procedure utilized the entire SED matrix as a classifier. In other words, all the relations between genes were considered in the classification. To determine whether the inclusion of the whole matrix was actually required to achieve the current accuracy, we investigated the efficacy of using single gene's SED as classifiers.

**Figure 1:**
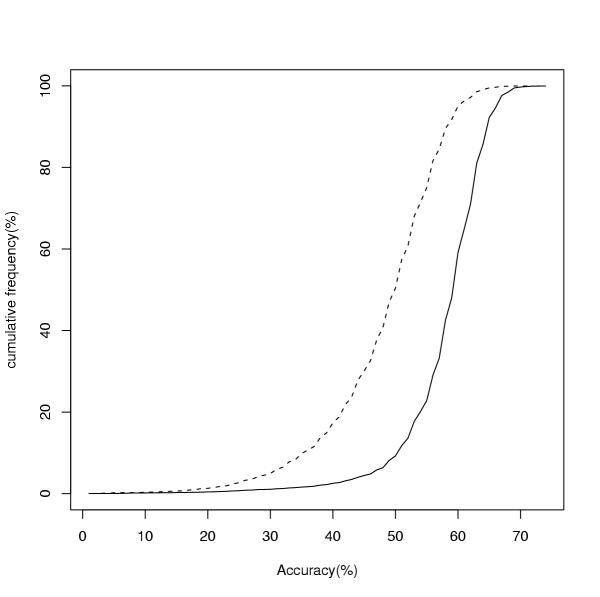
**Cumulative Frequency of Single Gene SED classifiers versus cross validation accuracy. **The X axis represents the cross validation accuracy and the Y axis represents the cumulative frequency of the single gene based classifiers. For each point (x,y) on the curve, y equals the percentage of classifiers that have an accuracy less than or equal to x. Solid curve – NN method with cutoff σ^2 ^= 0.05. Dashed line – NB method with σ^2 ^= 0.06.

**Figure 2:**
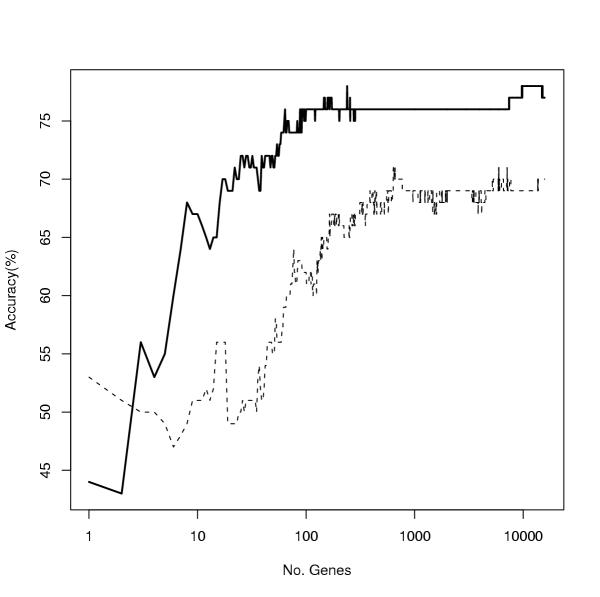
**Evaluation of the number of genes required to achieve the highest accuracy. **The X axis represents the number of genes used in the classifiers. The Y axis represents the cross validation accuracy. Solid line – NN method with cutoff σ^2 ^= 0.05. Dashed line – NB method with σ^2 ^= 0.06. X is in log scale. As X increases more genes are included in the classifier in the order they are represented in the original microarray (unselected for performance).

In these cases, we define a classifier based on gene i and its relative expression to every other gene. Therefore, the score_i_(T, C) = Σ_j = 1,...,N _log(p_ij_), where p_ij _is as same as mentioned previously. Similarly, matches_i_(T, t) = Σ_j = 1,...,N _δ(s_ij_^T^, s_ij_^t^) defines a classifier based on gene i's SED. Fig. 1 shows a display of the cumulative frequency of single gene SED based classifiers versus accuracy for all the 16063 classifiers. In general, single gene-based classifiers performed worse than the whole genome-based classifiers, as expected. Nevertheless, most of the classifiers performed reasonably well, compared with just using single gene expression levels. About 80% of the single gene based classifiers resulted in an accuracy between 40% and 60%, while about half of the classifiers had an accuracy greater than 50%. These results suggest that there is a lot of redundant information in the SEDs and SED probabilities and that our method should be reasonably robust. We then investigated the number of genes that are required to achieve the current accuracy. Fig. 2 shows the combined results for classifiers using only a subset of genes. Our results suggest that a subset of genes (~200) is sufficient for predictions and that the prediction accuracy is stable after 1000 genes.

### Different classification accuracy between tumor classes

From the analyses described above, we noticed that there was a significant difference in accuracy between different tumor classes. For 3 classes (LY, LE, CNS) we obtained either 100% or close to 100% accuracy (see Table 2 for detail). Since these happen to correspond to the 3 classes with more than double the number of training samples than the other classes, we tested whether this high accuracy is due to the larger sample sizes by using only 8 training samples for every tumor classes. The results were essentially the same, indicating that sample size is not the issue. On the other hand, there are classes where we obtained very poor results; these often happen to be the same classes where SVM in [19,20] performed poorly as well (see Table 2).

**Figure 3:**
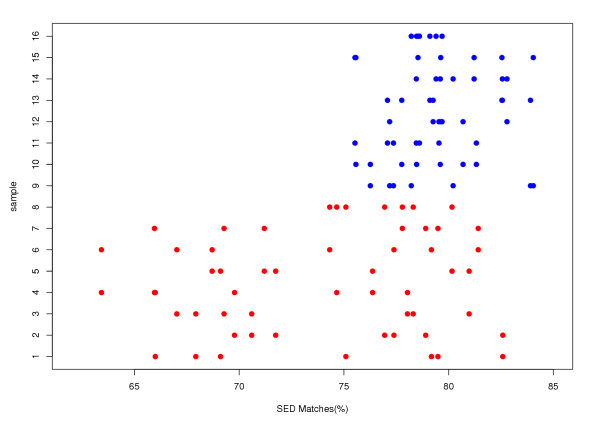
**Scatterplot of SED Match scores of OV and CNS samples. **The X axis represents the SED Match scores from eq. (3) / N^2^, where N = 16063, the total number of genes. The Y axis represents the sample id. Samples 1–8 (shown in red) are from class OV while 9–16 (shown in blue) are from class CNS. Only 8 samples from the CNS class were used for ease of comparison. Other samples gave similar results. For each sample, its Match scores / N^2 ^against all the other 7 samples within the class are shown. The Match scores of OV class are much more dispersed (to the left), compared with that of CNS class.

We were interested in exploring the possible reasons for misclassification. In Fig. 3, a scatterplot of the SED Match scores (without feature selection) between 8 OV samples and 8 CNS samples is displayed. The OV and CNS class were selected since one is "very hard" and the other is "very easy" to classify. Without trying to be statistically correct, the plot does suggest that samples of the OV class are in general "farther away" from each other, compared with those from the CNS class. As this may be one of the reasons that the OV class is harder to classify, algorithms that take this kind of information into account may perform better than the simple ones we have presented here.

## Discussion

Although we used the multi-class tumor classification problem as our test case, our major goal was to illustrate the feasibility of the normalization free SED approach, and not in sample classification *per se*. Therefore, we chose the algorithms NB and NN for their simplicity and not for their performance in solving this specific problem. The performance of a classifier depends, in this case, mainly on the power of its algorithm, and the data representation it used. From a machine learning perspective, one can simply view the intensity -> SED transformation as a change in data representation, a mapping from the gene's attribute, intensity x, to some features SED. It was our goal to demonstrate that the new features (SED and SED probability), in addition to being normalization free, still convey the essential information in the original attribute, the intensity x.

Since the data representation is quite different between the intensity x (a real valued quantity) and SED (a binary valued vector of rather large size N), it is difficult to directly compare the two. No obvious yet non-trivial algorithms work with both representations; even if there were such an algorithm it is not clear that it would be the right one to use for comparison as it might well be the case that different data representation works best with different algorithms. Here, we have limited the presentation to some empirical results with SED representation, which are comparable with results using several different algorithms that are based directly on raw intensity [19,20]. Our classification results are close to those obtained with WV and kNN methods, which are based on directly focusing on intensity levels. Previous results using SVM were significantly better, but we feel the differences are due more to the power of the algorithm [24] than the way information is coded. In fact, slightly more accurate results are obtained with modification of algorithms that directly manipulate intensity levels [25,26]. We do not imply that the algorithms (NB and NN) we chose are better than other alternatives (and we do not have empirical evidence pointing either way). Instead, we fully expect more sophisticated algorithms would work better with the SED approach as well.

Certainly, SED probability is more information rich than SED. We expect that an SED probability based analysis would perform better than the simple binary valued SED. In this paper, we mainly tested the SED. SED probability is only used for a group of samples, not for single samples. If one limits oneself to use only raw data, then for single arrays one can only get SED. However, if some assumptions about the patterns of gene expression levels can be made, one can certainly get an estimation of SED probability even for a single array. For example, as in some nonlinear normalization algorithms, if one assumes that the variation of expression levels are similar for genes with similar expression levels, then one can estimate SED probability from a probability model. Also, the magnitude of the intensity difference can also be used to help such an estimation. Alternatively, as more and more microarray data become available, one can use other similar samples to get an estimation of a prior SED probability, and then use a Bayesian approach to estimate the sample's SED probability.

The obvious disadvantage of our SED based approach is that for each gene expression level, one is not dealing with a single real number but instead a vector of size N, where N is in the tens of thousands. This could significantly increase both computing time and memory requirement (however, see methods for details) On the other hand, it also has certain advantages: 1) It is free of normalization noise. Since it is generally believed that biological variation is larger than technical variation and normalization noise is just another source of technical variation, the benefit here is only of a limited scope. However, it may be important when the expression level difference one is interested in is small. 2) In addition to being normalization free, SED and SED probability also have the advantage in being distribution free, and therefore could perform better if the intensity levels were non-normal. 3) SED and SED probability are easier to interpret. SED values can easily be checked against raw intensity levels according to Eq. (1). While SED probability is one step further away from intensity levels, one could still have an intuitive sense of it and make comparisons between different experiments. It would be much harder to have a real grasp of the absolute gene expression level, except that it is "high" or "low" or somewhere "in between"; it is certainly harder to compare between experiments intuitively.

We have only tested the SED approach on datasets that are from the same chip format. Data from different chip formats or complete different technology platforms, of course, would be harder to compare. But they are also challenge for normalization based method. It would certainly be interesting to compare SED and normalization based method under these more challenging conditions.

If this normalization-free approach (SED) proves to retain the essential biological information in general, its application may be extended to meta-analysis where different datasets could be integrated and intervalidated. The method could also be used when the number of arrays is a limiting factor for experiments. For example, one could take advantage of the massive amount of public array data, obtain prior distribution of SED probabilities from datasets with similar conditions, and analyze new data within a Bayesian framework. If the performance of the nearest neighbor method in general is anywhere close to what we demonstrated in the multi tumor classifications here (as is clear from Eq. (3) and Fig. 3, the nearest neighbor method, without feature selection at least, allows direct sample vs. sample comparison. Note also that the samples in the multi tumor problems are from different biological specimens, therefore, large between-sample-variation is expected), it might be used as a microarray database query method, *i.e.*, to find similar microarray results in the database that are "similar" to one's own, independent of array annotations.

It might also be worth noting that the SED approach could easily be applied to other kinds of comparative data analysis for samples with very large numbers of "noisy" attributes. The SED approach may also perform better when between-sample-variation is large, especially if such variation contains some rather uninteresting technical measurement errors that would not affect within-sample-variations.

## Conclusions

We have proposed a new approach to analyze microarray data and tested the method on a set of publicly available datasets. The results were comparable to those obtained with some widely used normalization based algorithms. We hope that we have demonstrated that this normalization free method is feasible and promising. We think the SED based, normalization free approach could be used to complement the more popular normalization based approaches in microarray data analysis.

## Methods

Microarray data for multiple tumor samples were downloaded from . Naive Bayes and Nearest Neighbour Classifiers were implemented in the Java programming language. *Ad hoc *analysis was done with perl scripts. Graphics were generated using the R computing environment.

### Naive Bayes method

Because of the uneven and relatively small sample sizes for each tumor class (mostly 8 but up to 24), extra care was taken in computing sp_ij_. Assuming a prior probability of 0.5, sp_ij _was estimated by Bayesian posterior probability (m+1)/(n+2) where n is the total number of samples in the class and m is the total number of samples where x_i _> x_j_. For classes that were over-represented (sample size > 8), the threshold of sp_ij _was set to [0.125, 0.875], since the NB method is sensitive to the extreme values of sp, and samples can be over-predicated without thresholds.

In addition, several alternatives were tested to demonstrate that our results were reasonably robust and not sensitive to the particular choices we made:

1) To examine the influence of the sample size, in a separate analysis the sample size of ME, LE, CNS class was artificially reduced to 8, *i.e. *only the first 8 samples were used to calculate sp_ij _with no significant change of results observed;

2) Since sp_ij _depends on the sample size n for each tumor class, we have applied a "sample replacement" strategy in addition to the usual "take-one-sample-out" approach for cross-validation, *i.e. *when one sample is taken out as the test sample, another sample from the same class is duplicated to take its place to keep the sample size constant. Essentially the same results were obtained. Results reported are from the sample replacement runs.

In Feature Filtering, the variance of sp_ij _between all 14 classes was calculated as:

σ^2 ^= (Σ_C = 1,...,14,_sp_ij_^C ^* sp_ij_^C^)/14 - ((Σ_C = 1,...,14 _sp_ij_^C^)/14)^2 ^    (4)

with the test sample taken out, and used as the criterion for feature exclusion.

### Nearest neighbor method

Feature filter was done as in the Naive Bayes Method.

### Software implementation and availability

The analysis was done on a computer (Pentium M 1.5 GHz) operating under Microsoft XP. Both Naïve Bayes and Nearest Neighbor Classifiers are implemented in Java. Since SED can be easily calculated from the raw intensities only the later are kept in memory and SED are computed from the intensities on an as-needed bases. Memory needed to analysis the 144 samples is less than 64 MB.

The most computationally intensive algorithm that we tried is the Nearest Neighbor method without any feature selections and it takes about 10 sec to calculate SED score for one pair of tumor samples with about 16000 genes.

A Java program named SED (including source code) to perform nearest neighbor analysis of microarray samples is freely available by contacting author at hw14@columbia.edu.

## Authors' contributions

H.W. conceived of the SED study and performed implementations. H.H. refined the approach and provided additional statistical insight on SED. Both authors read and approved the final manuscript.

## Figures and Tables

**Table 1:** The relationship between accuracy (%) and the filter threshold The cross validation accuracies with Naive Bayes (NB) and Nearest Neighbor (NN) Methods are displayed with different filter cutoffs. The percentages of the features (gene pairs) used are listed as well. Since the number of features used are slightly different for different test samples, ranges are shown.

Filter Threshold	No Filter	0.02	0.04	0.05	0.06	0.08
NB	63	66	68	69	70	68
NN	70	73	75	77	75	74
Features used (%)	100	41–43	12–13	4.9–5.9	1.7–2.1	0.12–0.17

**Table 2:** Summary of cross validation results. All 14 tumor types and their abbreviations are listed in the first column. The sample sizes for each tumor type and the number of successfully classified samples by the SVM algorithm (from ref. [19]), the NB (Naive Bayes) algorithm with cutoff σ^2 ^= 0.06 and the NN (Nearest Neighbor) algorithm with σ^2 ^= 0.05 are listed.

Tumor type (Abbreviation)	Sample Size	SVM [19]	NB	NN
Ovary (OV)	8	3	1	4
Lung (LU)	8	4	1	3
Bladder (BL)	8	5	7	5
Melanoma (ML)	8	5	6	6
Renal (RE)	8	5	5	6
Pancreas (PA)	8	5	6	6
Colorectal (CR)	8	6	1	3
Prostate (PR)	8	6	6	6
Breast (BR)	8	7	3	4
Uterus (UT)	8	7	6	6
Mesothelioma (ME)	8	8	6	6
Lymphoma (LY)	16	16	14	16
Central Nervous System (CNS)	16	16	16	16
Leukemia (LE)	24	24	23	24
